# Adverse childhood experiences are associated with increased overdose risk in predominately Latinx adults seeking treatment for substance use disorders

**DOI:** 10.3389/fpsyt.2022.987085

**Published:** 2022-12-15

**Authors:** Cynthia A. Tschampl, Melisa Canuto, Diliana De Jesús, Melinda D'Ippolito, Micaurys Guzman, Mary Jo Larson, Emily Stewart, Lena Lundgren

**Affiliations:** ^1^The Heller School for Social Policy and Management, Brandeis University, Waltham, MA, United States; ^2^Casa Esperanza, Inc., Roxbury, MA, United States; ^3^Graduate School of Social Work, University of Denver, Denver, CO, United States

**Keywords:** adverse childhood experiences, Latinx, opioids, overdose, post-traumatic stress disorder, Puerto Rican, substance use disorder, SUD treatment

## Abstract

**Introduction:**

Almost no previous studies explored the relationship between adverse childhood experiences (ACEs) and overdose risk for individuals with substance use disorders (SUDs), and these did not focus on a Latinx population. This study examined the relationship between ACEs, reporting PTSD symptoms, and lifetime experience of overdose in a sample (*n* = 149) of primarily Latinx adults seeking treatment for substance use disorder (SUD).

**Materials and methods:**

Administrative data from an integrated behavioral health and primary care treatment system in Massachusetts were analyzed through bivariate analyses and multiple logistic regression. The final model examined the association between self-reported ACEs, PTSD screen, and lifetime drug overdose. We controlled for demographic characteristics and heroin use and explored alternative measure specifications.

**Results:**

ACEs scores were high with 58% having experienced 4+ ACEs. Female gender was associated with a 24% higher ACE score than male gender (*p* < 0.01). In the multiple logistic model each additional ACE was associated with 1.3 times greater odds of overdose (*p* < 0.01). Those reporting heroin use had 8.8 times greater odds of reporting overdose compared to those reporting no heroin use (*p* < 0.001). Gender, age, Puerto Rican ethnicity, years of cocaine use, receiving public assistance income, and a positive initial PTSD screen were not significant. Findings were robust in sensitivity testing.

**Discussion and conclusion:**

We found the number of ACEs and reported heroin use significantly and positively associated with self-report of overdose in both bivariate and multiple logistic regression analyses. In contrast, a positive initial screen for PTSD was only significantly associated with overdose in the bivariate analysis. Increased screening for ACEs is warranted and ACE-specific treatment is suggested for SUD treatment programs offering trauma-informed services for adults.

## 1. Introduction

Opioid overdose surpassed homicides as the leading cause of injury-related death in the US in 2015 (1). Further, the age-adjusted drug overdose death rate in the US remains at an all-time high, at 21.7 per 100,000, and natural, synthetic, and semisynthetic opioid-related overdoses are increasing (2, 3). Fatal overdoses are not as frequent as non-fatal overdoses (4), which can lead to cognitive, cardiac, and other long-term impairments (5).

Risk factors for overdose include drug dose, mental health disorders, and substance use disorders (SUDs) (6). In a large California health plan, over 7.2% of patients with a non-fatal overdose had a repeat overdose in a 12-month period, with greater risk of repeat overdose among those with opioid use disorder (7). Because national-level data (and other high-level aggregate estimates) on drug use can obscure higher-risk subgroups (8–11), it is important to identify geographically and culturally specific risks for overdose to best serve the local population.

For example, in 2018 the Commonwealth of Massachusetts had a rate of 29.3 opioid-related deaths per 100,000, twice the national rate (12). A major cause of overdose in MA is street drugs cut with fentanyl (13). Fentanyl is 80 to 100 times as potent as morphine (14) and contributes to unintentional overdose. Additionally, while the Latinx population comprised 4% of all annual deaths in the Commonwealth, they comprised 13% of confirmed opioid-related deaths in 2019 (15). Moreover, the rate of overdose deaths involving stimulants and opioids in Massachusetts is currently highest among Latinx residents (12.3 per 100,000), an increase of 36% per year from 2012 to 2018 (16). This trend is consistent with the greater number of barriers to care Latinx people face, e.g., cultural factors, perception of need, stigma, and logistics (17–19).

Adverse childhood experiences (ACEs) are defined as negative events that occur before a child reaches the age of 18 and may cause trauma (20). The ACEs questionnaire asks about various types of childhood abuse (psychological, physical, and sexual) and household dysfunction (divorce, inter-partner violence, substance use, mental illness, and criminal behavior) (21). The ACEs that are trending upward in the U.S. include parental problematic alcohol use (2000–2016) and parental problematic drug use (2005–2016) (22).

A seminal study on ACEs and problematic drug use found ACEs to be the dominant attributable risk factor for illicit drug use, drug use disorders, and parental drug use (23). Subsequent research affirmed a strong association between ACEs and substance use problems, either as specific experiences (24–26), a dose response, (27–29) or both (27, 30). Other studies focused on populations with SUD and found individuals who reported ACEs were overrepresented (31–35) and had greater severity of childhood maltreatment than the general population (36). Bórquez-Infante and colleagues identified ACEs as a risk factor for psychotic episodes among patients with SUD (37). However, we found only one study that looked at the relationship between ACEs and overdose in a population with SUD (38). Specifically, Stover and colleagues found higher mean ACE scores among two subgroups who reported suicide ideation and/or suicide attempts in addition to intentional or unintentional overdose compared to those who reported only unintentional overdose (38).

Existing research also suggests individuals with post-traumatic stress disorder (PTSD) are overrepresented in populations with SUD (35) and that these disorders are frequently co-morbid (39, 40). Schneider et al. (41) found PTSD is common among vulnerable populations who have experienced overdose. A Canadian study (42) identified a strong, significant association between a provisional PTSD diagnosis and overdose. Fendrich et al. found PTSD was significantly associated with recent overdose (in the past three months) (43).

The study presented here is testing the hypothesis that both ACEs and reporting PTSD symptoms are significantly and independently associated with reporting one or more overdoses in a primarily Latinx population entering treatment for substance use disorder (SUD).

## 2. Materials and methods

This cross-sectional study presented here examined the relationship between self-reported ACEs, PTSD symptoms, and lifetime experience of overdose in a sample (*n* = 149) of primarily Latinx adults entering treatment for an SUD. The study controlled for demographic and socioeconomic characteristics and lifetime heroin and cocaine use prior to intake. Sensitivity analyses tested alternative specifications for key concepts.

### 2.1. Sample

The sample included 149 adults who entered SUD treatment disorder between 2019 and 2020 in the Commonwealth of Massachusetts. The sample included all those who entered residential and outpatient treatment of Casa Esperanza, Inc. (Casa) as part of receiving SAMHSA funded treatment services. Casa patients enter treatment through three main pathways. The most common situation is referral through external sources (e.g., prisons, detoxification centers, social service agencies, health care providers, police, and emergency rooms). However, Casa also conducted community outreach in area hot spots, and many patients independently contacted Casa because they heard about the program from either current or previous participants.

The clients completed a detailed administrative interview that included all required questions for the U.S. Government Performance and Results Act (GPRA) and the National Outcomes Measures (NOMs) (44), as well as additional behavioral health validated scales. All study interviews were conducted in either English or Spanish by multilingual and multicultural program staff, all of whom were trained to administer the interview tool by the same evaluation staff member. Program staff took care to build rapport with clients in culturally competent ways, which is particularly important since the interview included many personal and sensitive questions. Casa staff read the standardized questions to clients and noted client responses. This generated the data for all variables used in this study, except for the substance use diagnoses. The SUD diagnoses were extracted by Casa staff from their electronic health record (EHR).

The study was reviewed and approved as exempt by both the University of Denver and Brandeis University Institutional Review Boards. Only baseline data were used in the study presented here.

### 2.2. Treatment organization from which the sample was drawn

The Casa Esperanza, Inc. behavioral health system is the Commonwealth's largest multicultural and multilingual substance use disorder treatment organization focused on the Latinx population. Casa offers short term and long term (up to a year) residential treatment, outpatient, acute care, supportive housing, and crisis stabilization services. Casa's integrated, co-located behavioral and primary health care services are available to both residential and outpatient clients. These services include many evidence-based practices supportive of behavioral health, medical health, social determinates of health, and overall wellbeing.

### 2.3. Outcome variable

The outcome variable of interest was self-report of having a history of one or more overdoses. Because multiple drugs are often detected in patients who overdose (15, 38, 44–46), we did not disaggregate by substance involved in overdose. Self-report for drug use experience has been found to be generally reliable (47, 48).

### 2.4. ACEs variable

ACEs were measured using the original CDC-Kaiser ACE scale (Wave 2) developed by Felitti et al. (21, 49). During their administrative interview, clients were asked yes-no questions regarding the ten ACEs in three domains on the scale (abuse, neglect, and household disfunction) and Casa staff noted the answers. The “ACEs score” measure was summative with one point given for each affirmative answer, for a maximum score of ten. This original ACE scale is a reliable and valid retrospective assessment of negative experiences before age 18 with good test-retest reliability (Kappa = 0.51–0.95) (50, 51) and internal consistency (Cronbach's alpha = 0.88) (32, 52). In this sample we tested for internal reliability given several administrative staff were conducting the client interviews and found our Cronbach's alpha = 0.78, which is considered a good level. For the purposes of sensitivity analyses, we also created an alternative specification—a dummy variable of reporting four or more ACEs vs. reporting fewer than four. We chose this cutoff because of the balanced distribution in our sample and based on prior literature (27, 32, 50, 53–56).

### 2.5. Control variables

PTSD symptoms were measured using the SAMHSA-required GPRA PTSD screening tool (45) *via* self-report during the same administrative interview described above. The tool begins with a question regarding a possible precipitating event, “Have you ever experienced violence or trauma in any setting (including community or school violence; domestic violence; physical, psychological, or sexual maltreatment/assault within or outside of the family; natural disaster; terrorism; neglect; or traumatic grief)?” If a client answered “yes”, then they were asked the following, “IF YES: Did any of these experiences feel so frightening, horrible, or upsetting that, in the past and/or the present, you: 1. Had nightmares or thought about the trauma when not wanting to; 2. Tried hard not to think about the trauma/made special efforts to avoid situations that remind of the trauma; 3. Constantly on guard, watchful, or easily startled; 4. Felt numb and detached from others, activities, or surroundings?” These questions represent four of five questions from the Primary Care PTSD Screen for DSM-5, or PC-PTSD-5 (57), which has been found to be an effective and efficient screening tool for PTSD primary care clinics for a veteran population (58). We used the definition of a positive initial screen for PTSD, as answering “yes” to three or more of the four symptom category questions and an alternative definition of answering “yes” to two or more for sensitivity analysis. Those with a code of “zero” included both those who answered “no” to having a precipitating experience of violence or trauma and those who answered “yes” to having experienced a precipitating event but then “no” to all four PTSD symptom category questions. In this sample we found an excellent level of internal validity (Cronbach's alpha = 0.91).

Substance use variables: We constructed several variables relating to problematic substance use. Three dichotomous variables indicated whether the client's primary substance use diagnosis was related to opioids, cocaine/crack, or alcohol. We also created two continuous variables indicating the total number of years of lifetime heroin and cocaine use, as reported by clients in response to the federal GPRA interview questions, specifically: “How many years in your lifetime have you used heroin?” and “How many years in your lifetime have you used cocaine or crack?” First, the longer a person has used drugs the more opportunities for overdose are incurred. Second, among the study sample, heroin and cocaine were highly prevalent. Third, overdoses involving simulants such as cocaine are on the rise (16). Finally, self-reported substance use among adult users has been found to be reliable (47, 48).

Gender: this variable was coded as a dichotomous variable: male and female. The reason this variable was included is that, in the past decade, there has been an overall large increase in non-fatal overdose and deaths that was significantly greater among US males compared to women (59, 60).

Sexual identity: we coded this variable as a dummy of heterosexual vs. not heterosexual. However, there was little meaningful variation in our sample, so it was not included in any of the multiple logistic models.

Age: this variable was coded as a continuous variable. Research has identified younger age appears to be associated with an increased risk of fatal overdose and an increased number of drug related deaths among young unexperienced users of opioid over the last decade (1, 61).

Formal education level: we coded education as a dummy variable of fewer than 11 years of formal education vs. 12 or more years, including obtaining the equivalent of a high school diploma. Education has been found to be associated with overdose (62).

Latinx ethnicity: individuals in the U.S. who identify as Puerto Ricans have a higher rate of use of opioids (primarily heroin and ketamine), high injection rates, and high rates of HIV related to drug injection compared to other Latinx groups (63, 64). Other studies also show patterns of drug use and risky drug use practices differ between and within Latinx groups requiring culturally competent addiction treatment providers (64, 65). We coded both Puerto Rican ethnicity and Latinx ethnicity as two separate dichotomous variables.

### 2.6. Statistical analysis

Univariate descriptive statistics were used to describe the research sample. Next, bivariate analyses were conducted using chi-square and logistic regression to examine the relationship between lifetime overdose, ACEs, and control variables. We then created a multiple logistic regression model that included variables significantly associated with overdose at the bivariate level, as well as demographic data typically deemed important to control for, i.e., gender and age. We conducted tests for multicollinearity, linearity with the logit, and goodness of fit and adjusted the model accordingly. Finally, we conducted sensitivity analyses using alternative specifications for ACEs, number of reported PTSD symptoms, and ethnicity, as well as a “kitchen sink” model that included all the sociodemographic variables.

There were relatively few missing data. There were only two values of “don't know” in the PTSD symptom data (out of 5 × 149 = 745 values). They were both interpolated to be “no.” There were 19 missing values in the ACEs scale (out of 10 × 149 = 1,490 values). One client refused to answer all ten ACEs, and a second client refused seven of ten ACEs questions; we coded their ACE scores as missing, meaning their cases would be dropped during regression analyses. Two other clients refused to answer one question each, and so their ACE score was interpolated to be the sum of their nine answers. Therefore, they were included in all analyses, except the valid percent calculations. There were six (of 149) missing values for primary substance use diagnosis, all of which were interpolated based on self-report lifetime drug use data. All remaining variables had a 100% response rate.

Statistical analyses were conducted using SPSS software version 27 (IBM Corp 2020); the level of statistical significance considered was *p* < 0.05.

## 3. Results

### 3.1. Sample description

Table 1 shows the sample characteristics in total and disaggregated by self-report of any lifetime overdose. In this sample, 58 (39%) reported one or more overdose. The mean age was 42 years (SD 9.7), and the majority (73%) were males. While 87% identified as Latinx, 60% (89/149) identified specifically as Puerto Rican. Of the remaining non-Puerto Rican Latinx clients, there were 20 Dominicans, 17 Central Americans, four South Americans, two Mexican, one Cuban, one Cape Verdean, and one mixed Puerto Rican-Korean. Additionally, two refused to specify further.

**Table 1:** Sample description, *n* = 149.

	**History of overdose**	**Never overdosed**	**Entire sample**	***P*-value**
	***n* = 58**	***n* = 91**	***n* = 149**	
**Sample characteristics**	***n*** **(%)**	***n*** **(%)**	***n*** **(%)**	
**Gender**				*p* = 0.25
Male	39 (36.1)	69 (63.9)	108 (72.5)	
Female	19 (46.3)	22 (53.7)	41 (27.5)	
Latinx ethnicity	48 (36.9)	82 (63.1)	130 (87.2)	*p* = 0.29
Puerto Rican ethnicity	43 (48.3)	46 (51.7)	89 (59.7)	*p* < 0.01
**Sexual identity**				*p* = 0.08
Heterosexual	58 (40.8)	84 (59.2)	142 (95.9)	
Other	0 (0.0)	6 (100)	6 (4.1)	
**Education**				*p* = 0.08
Has a high school diploma, equivalent, or schooling beyond high school	39 (44.8)	48 (55.2)	87 (58.4)	
Has 0–11 years of formal education	19 (30.6)	43 (69.4)	62 (41.6)	
Employed either part time or full time	4 (26.7)	11 (73.3)	15 (10.1)	*p* = 0.46
Has income from public assistance	34 (47.9)	37 (52.1)	75 (50.3)	*p* = 0.03
Spent most of the past 30 days on the street or in a shelter	28 (43.1)	37 (56.9)	65 (43.6)	*p* = 0.36
**Behavioral health items**				
Primary SUD diagnosis of opioid use disorder	48 (60.0)	32 (40.0)	80 (53.7)	*p* < 0.001
Primary SUD diagnosis of cocaine use disorder	7(29.2)	17 (70.8)	24 (16.1)	*p* = 0.40
Primary SUD diagnosis of alcohol use disorder	2 (4.8)	40 (95.2)	42 (28.2)	*p* < 0.001
Reported heroin use	53 (55.2)	43 (44.8)	96 (64.4)	*p* < 0.001
Reported 4 or more ACEs	42 (49.4)	43 (50.6)	85 (57.8)	*p* < 0.01
Reported 3–4 PTSD symptom categories	37 (49.3)	38 (50.7)	75 (50.3)	*p* = 0.01
Reported 2–4 PTSD symptom categories	42 (47.7)	46 (52.3)	88 (59.1)	*p* = 0.01
	**Mean (SD)**	**Mean (SD)**	**Mean (SD)**	
Total ACEs score, 1–10	5.1 (2.8)	3.3 (2.5)	4.0 (2.7)	*p* < 0.001
Age, years	41.3 (9.2)	42.6 (9.9)	42.1 (9.7)	*p* = 0.40
Lifetime use of heroin, years	13.5 (10.5)	6.0 (10.4)	8.9 (11.0)	*p* < 0.001
Lifetime use of cocaine, years	12.2 (10.8)	7.6 (9.9)	9.4 (10.5)	*p* = 0.01

ACE, adverse childhood experience; PTSD, post-traumatic stress disorder; SD, standard deviation.

Six percent identified as other than heterosexual, and the majority (58%) had obtained at least a high school diploma (or equivalent). Fifteen (10%) were employed either full- or part-time, while half received at least some public assistance income. Forty-four percent reported spending most of the past 30 days on the street or in a shelter.

Half reported three or four of the PTSD symptom categories and 59% reported 2–4 PTSD symptom categories. The mean number of years of lifetime heroin use was 8.8 (SD 11.0), while that for cocaine was 9.4 years (SD 10.5). The primary SUD diagnosis was opioid use disorder (OUD) for 54%, cocaine use disorder for 16%, and alcohol use disorder (AUD) for 28% of the sample. The remaining three clients had a primary substance diagnosis dealing with marijuana (data not shown).

The sample reported high levels of ACEs. Table 1 shows the overall average number of ACEs reported was 4.0 (SD 2.7). Table 2 shows the frequency of individual ACEs. The most often reported ACEs were living with someone with alcohol or drug problems and experiencing divorced parents. Even the least frequently reported ACE, sexual abuse, was experienced by more than one-fifth of respondents (21%). Eighty-five people (58%) reported four or more ACEs. Only 17 (12%) reported no ACEs (data not shown).

**Table 2:** Sample exposure to adverse childhood experiences (ACEs), *n* = 149.

**ACE question**	**Yes**	**Valid percent**
Did a parent or other adult in the household often swear at you, insult you, put you down, or humiliate you? Or act in a way that made you afraid that you might be physically hurt?	71	48.3
Did a parent or other adult in the household often push, grab, slap, or throw something at you? Or ever hit you so hard that you had marks or were injured?	63	42.9
Did an adult or person at least 5 years older than you ever touch or fondle you or have you touch their body in a sexual way? Or try to or actually have oral, anal, or vaginal sex with you?	31	21.1
Did you often feel that no one in your family loved you or thought you were important or special? Or your family didn't look out for each other, feel close to each other, or support each other?	53	35.8
Did you often feel that you did not have enough to eat had to wear dirty clothes and had no one to protect you? Your parents were too drunk or high to take care of you or take you to the doctor if you needed it?	40	27.0
Were your parents ever separated or divorced?	90	62.1
Was your mother or stepmother often pushed had something thrown at her or sometimes or often kicked hit with a fist or hit with something hard or ever repeatedly hit over minutes or threatened with a gun or knife?	39	26.5
Did you live with anyone who was a problem drinker or alcoholic or who used street drugs?	90	60.8
Was a household member depressed or mentally ill or did a household member attempt suicide?	61	41.5
Did a household member go to prison?	55	37.4

### 3.2. Bi-variate findings

Table 1 depicts the bivariate correlates of reporting overdose. Ethnic identification as Puerto Rican was significantly associated with overdose (positive direction, Pearson Chi-square *p* < 0.01). Additionally, total ACE score (*p* < 0.001), an initial positive screen for PTSD as an adult (*p* = 0.01), lifetime years of heroin use (*p* < 0.001), and lifetime years of cocaine use (*p* = 0.01) were all positively associated with increase in overdose risk. Reporting some public assistance income was also significantly associated at the *p* < 0.05 level. A primary SUD diagnosis of either OUD or AUD were both significantly associated with reporting overdose at the *p* < 0.001 level. In contrast, none of the following variables had a significant association with overdose: age, gender, Latinx ethnicity (all subgroups combined), sexual identity, education, employment, living in a shelter during the past 30 days, and primary SUD diagnosis of cocaine use disorder.

We further explored bivariate relationships between the ACEs and PTSD symptoms variables and the other control variables (Supplementary Table 1). Both specifications of reporting a positive initial PTSD screen (*p* < 0.001) and female gender (*p* < 0.01) were all significantly associated with having a higher ACEs score. Being female was associated with a 24% increased ACE score compared to male gender and was also significantly and positively associated with reporting 3–4 PTSD symptom categories (Pearson Chi-square *p* < 0.01). Additional bivariate findings are included in Supplementary Table 1.

### 3.3. Tests for linearity and multicollinearity

We ran a series of tests to account for the assumptions related to logistic regression. No problematic multicollinearity was detected; indeed, all variables, including ACEs and PTSD symptoms variables, passed the most conservative common threshold of a variance inflation factor (VIF) <3. We did, however, find our variable of self-reported “lifetime years of heroin use” violated the assumption of linearity of the logit using the Box-Tindwell test. Therefore, we tested additional transformations of the variable, but they likewise failed the Box-Tindwell. We tested a dichotomous version of the variable, i.e., reported any heroin use vs. not, and found no violations of the assumptions for logistic regression. The three descriptive variables indicating the primary SUD diagnosis were also found to be in violation of the linearity with the logit assumption, and so were not used.

### 3.4. Multiple logistic regression findings

Table 3 presents our final multiple logistic regression model. For each additional ACE reported, the likelihood of reporting at least one overdose was 1.3 times the odds greater (*p* < 0.01). Additionally, clients who reported heroin use had 8.8 time the odds (*p* < 0.001) of reporting overdose compared to those with no reported heroin use. Reporting 3–4 PTSD symptoms (*p* = 0.44), Puerto Rican identity (*p* = 0.18), lifetime years of cocaine use (*p* = 0.19), and public assistance income (*p* = 0.035) became not significant in the final model. As expected, age and gender were not significantly associated with overdose. The final model passed the Hosmer-Lemeshow goodness-of-fit test and showed a Nagelkerke R Square of 0.41.

**Table 3:** Multiple logistic regression model on lifetime overdose, *n* = 147.

**Independent variable**	**Adjusted odds ratio**	**95% CI lower**	**95% CI upper**	***P*-value**	**VIF**
Total ACE score, 0–10	1.27	1.07	1.51	< 0.01	1.36
Reported 3–4 PTSD symptom categories vs. did not	1.45	0.57	3.71	0.44	1.41
Reported heroin use vs. did not	8.84	2.89	27	< 0.001	1.43
Age	0.96	0.93	1.02	0.17	1.36
Female vs. male	0.78	0.26	2.29	0.65	1.39
Puerto Rican identity vs. not	2.1	0.72	6.14	0.18	1.54
Reported public assistance income	1.56	0.62	3.93	0.35	1.35
Number of lifetime years use of cocaine	1.03	0.99	1.07	0.19	1.24

ACE, adverse childhood experience; CI, confidence interval; PTSD, post-traumatic stress disorder; VIF, variance inflation factor (to test for problematic multicollinearity).

### 3.5. Sensitivity analyses

To test the robustness of our findings and how we measured key constructs, we conducted several sensitivity analyses. The findings from all the alternate models affirm our final multiple logistic model results (Supplementary Table 2). Furthermore, all models passed the Hosmer-Lemeshow goodness-of-fit test.

For example, we used the dummy specification of number of ACEs (i.e., four or more vs. 0–3 ACEs). Both the ACEs score and heroin use remained significant at the *p* < 0.05 and *p* < 0.001 level, respectively. The other five control variables remained not significant.

The second alternative model used 2–4 reported PTSD symptom categories as a positive initial screen instead of 3–4 reported PTSD symptom categories. This PTSD-related variable remained not significant, while the ACEs score and any heroin use remained significant at the levels of *p* = 0.01 and *p* < 0.001, respectively.

A third analysis controlled for all Latinx identities in aggregate rather than Puerto Rican identity specifically. Latinx ethnicity was not significantly (*p* = 0.32) associated with reporting overdose. Moreover, the level of significance for any heroin use was maintained at *p* < 0.001, and ACEs likewise continued to be significant at the *p* < 0.01 level.

Finally, we included all sociodemographic variables. In that multiple logistic regression, age, gender, 12 or more years of school, spending most of the past 30 days homeless or in a shelter, employment status, having public assistance income, years of cocaine use, Puerto Rican ethnicity, and reporting 3–4 PTSD symptoms were not significantly associated with overdose. An additional ACE reported was associated with 1.3 times increase in the odds of reporting overdose (*p* < 0.01) and reporting heroin use was associated with 9.3 times the odds of reporting overdose (*p* < 0.001), compared to those not reporting any heroin use.

## 4. Discussion

### 4.1. Findings in context

This examination of lifetime overdose among a predominantly Latinx treatment-seeking sample in Massachusetts found half of the sample had a positive initial screen for PTSD as an adult as well as a high level of self-reported adverse childhood events overall, which was significantly higher for those reporting female gender. At the bivariate level total ACEs score, positive initial screen for PTSD, Puerto Rican ethnicity, income from public assistance, heroin use, and years of cocaine use were all significantly associated with increased risk of overdose, In the multiple logistic regression, we found only the ACEs score and reported heroin use maintained that significance, yet both associations remained robust through rigorous sensitivity testing.

Despite some improving trends in ACEs in the U.S. (22), our sample had very high self-report of adverse childhood events. Only 12% had no exposure, whereas a national sample reported 60% of white non-Latinx children with no ACEs and 49% of Latinx children with no ACEs (66). The range in our sample went from 21% having experienced sexual abuse to 62% experiencing divorced parents. Fifty-eight percent of our sample reported four or more ACEs. This was smaller than the 83% in a small study among SUD treatment seekers (32), but larger than the 47% level in a similar sized study involving only Black males (29) and the 43% in a study with LGBT youth (55). Overall, it is consistent with the overrepresentation of ACEs in persons with SUD (31–35).

The bivariate analyses further revealed that while Puerto Rican identity was highly and significantly associated with increased risk of reporting overdose, the aggregate Latinx identity was not. This is consistent with the literature related to the “immigrant health paradox,” (also sometimes referred to as “Hispanic health paradox”) which describes immigrants having better health indicators such as lower chronic conditions (67) than US-born peers of similar sociodemographic status (68). Previous studies have also found that Puerto Ricans—compared to one or more other Latinx subgroups—have higher psychiatric disorders (69) and illegal drug use (10). Cano found that only Puerto Rican ethnicity superseded the 2017 age-adjusted drug overdose mortality rate among non-Hispanic Whites, out of six different Latinx sub-groups (8). Other research has found Puerto Ricans had poorer overall health, including psychological distress (70) and higher levels of SUDs (69), as well as higher use of opioids (primarily heroin and ketamine), injection rates, and rates of HIV related to drug injection compared to other Latinx groups (63, 64). Work such as that from Delgado and colleagues may further indicate some latent acculturation variables (71).

We found both constructs of a positive initial screen for PTSD were significantly associated with overdose at the bivariate level. This is consistent with prior research showing a strong relationship between PTSD and overdose (35, 39–42, 47, 49, 58). However, when ACEs, years of use of heroin, and Puerto Rican identity were controlled for in the final model, the positive initial screen for PTSD was no longer significant. It is possible the ambiguous timeframe and the reduction from five to four symptom-related questions built into the federal GPRA PTSD screening tool reduced our ability to find a significant association. This may also contraindicate the usefulness of adjusting validated tools without additional test-retesting.

Our final, multiple logistic regression model also showed heroin use had a very strong and positive association with overdose, which was expected since the majority of overdose deaths involve opioids (72, 73) and the heroin supply often contains fentanyl (74).

The other key finding that each additional ACE was associated with 1.3 times the likelihood of an overdose is consistent, though larger, than that found by Stein and colleagues (75). Possible explanations for the larger magnitude are the local context of drug market factors, use patterns, and healthcare patterns. Moreover, our sample was 60% Puerto Rican (87% Latinx) rather than white, which is consistent with a national sample of Latinx person reporting greater exposure to ACEs (66), as well as suffering from systemic racism.

Our sensitivity analyses produced remarkably robust findings regarding the association between ACEs, positive initial PTSD screen, and heroin use with history of overdose. Indeed, the novelty of this study are the strength and stability of both the findings that ACEs are strongly associated with overdose and that when the ACE score is included in the multiple logistic models, then a positive initial PTSD screen as an adult was no longer significant. We do not interpret this to mean prior studies linking PTSD and overdose are contradictory. Rather, our findings indicate adverse childhood experiences are both strongly associated with having PTSD symptoms as an adult, as well as independently associated with overdose. Mersky et al. found some evidence that adult adversity mediates the long-term consequences of ACEs (76).

Clinically speaking, this study underscores the need to screen for ACEs rather than just PTSD upon intake for SUD treatment programs endeavoring to provide trauma-informed services. This suggests people with high ACEs may need specialized services compared to those without such risk factors, which in turn may suggest improving referral pathways. For SUD treatment programs, this also could include hiring a staff psychiatrist and/or psychologist for assessing specific psychiatric disorders in addition to SUD and for treating both proximal and distal trauma. For example, Cloitre et al. conducted a randomized controlled trial and found skills training in affect and interpersonal regulation (STAIR) followed by supportive counseling resulted in full remission for PTSD due to childhood abuse compared to STAIR followed by exposure therapy (77). Schimmenti et al.'s findings suggested adding treatments that “aim to enhance adaptive affect regulation” (78). Dolbier et al. found the connection between ACEs and generalized anxiety decreased as dispositional mindfulness increased (79).

Our findings that higher ACEs scores were associated with lifetime overdose history suggest that further attempts to repeat these analyses would be worthwhile in a sample with greater ethnic and racial diversity, clear temporal relationships between PTSD symptoms and overdoses, and using a more rigorous assessment. We are also careful to note that the ACEs scale should not be used as a diagnostic tool for the purposes of treatment algorithms for individuals (80), but rather as a screening for evidence-based assessment tools and/or for population-level insights.

### 4.2. Limitations

There are seven limitations to consider. First, this was a cross-sectional study with a convenience sample. However, the sample represented all clients fully enrolled in SUD treatment at Casa during this period and offer a powerful glimpse into practice-based data from a community-based organization serving primarily Spanish speakers. Additionally, ten Casa staff collected these data from clients and no inter-rater reliability data were available; however, all staff received the same training from the same evaluation staff trainer, Casa staff read the same standardized questions in the same order, and just two of the staff completed the majority of the surveys.

While there is a strength to combining all types of SUDs and overdoses together—because most overdoses include more than one substance (44)—future studies are needed with larger, more generalizable samples to further test the relationship between ACEs, PTSD, and overdose.

We included the original Wave 2 CDC-Kaiser ACE scale in our study; however, others such as Finkelhor and colleagues found that added peer- and community-related violence questions increased the predictability of mental and physical health issues (81). A different extension is being developed by researchers such as Guo et al. who have started to include resiliency factors to their ACEs assessments (82). We support these developments but were constrained by the limits of working within the real-world workflow of a community-based organization.

Some portion of the overdoses in our sample may have been attempts at suicide, and ACEs have been found to be strongly associated with suicidal behaviors (38). For example, ACEs were found to be positively associated with lifetime attempted suicide reported during adulthood after adjusting for age, race/ethnicity, gender, marriage, and educational attainment (30). Individuals who reported experiencing emotional abuse during childhood had 5.59 times increased odds of reporting an attempted suicide (30), and sexual abuse was very prevalent in a sample of women hospitalized for a deliberate drug overdose (83). Nevertheless, these findings are complementary to our findings that show ACEs to be associated with overdose since overdose and suicide attempts have overlapping risk factors (38, 84).

Due to federal guidance, our data relied on a PTSD screening tool with an unclear timeframe, meaning we were unable to determine the exact temporal order of PTSD symptoms and overdoses. We did have partial data from this same cohort (46/149) who reported on overdoses in the past year, and only four had overdosed in the previous 12 months (data not shown). This and other contextual factors suggest the clients were referring to older overdoses and more recent PTSD symptoms. Nevertheless, future studies warrant clearer temporal data characterization.

Furthermore, our sample size of other Latinx ethnicities was relatively small. Additional research with larger subgroups of different Latinx ethnicities could provide critical insight for treating clinicians, especially if some subgroup identities are associated with higher or much higher risk (8). We were also limited in the demographic data we were able to present, for example, marriage status and race. However, while race—in addition to ethnicity—is usually seen as an important variable for analysis, it lacks salience in a special way among Puerto Ricans (85–87), who made up the majority of this treatment cohort. For example, Landale and Oropesa found that both Puerto Ricans on and off the island most often self-described their race as “Puerto Rican.” but for those who did not, Puerto Ricans living on the mainland identified their race as “Hispanic” or “American” (85). Indeed, the race question was banned from the decennial census distributed on the island for 50 years. Vargas-Ramos compared the race responses among Puerto Ricans living on the island (3.4 million) and those living on the mainland (3.8 million) for the 2,000 Census, when the question was reinstated. White was recorded for 81% of those on the island and only 47% of those on the mainland that year (86).

### 4.3. Conclusion

We found the number of ACEs and reported heroin use significantly and positively associated with self-report of overdose in both bivariate and multiple logistic regression analyses. These relationships held for all alternative models tested. In contrast, a positive initial screen for PTSD was only significantly associated with overdose in the bivariate analysis. Increased screening for ACEs is warranted and ACE-specific treatment is suggested for SUD treatment programs offering trauma-informed services for adults. Finally, the relationship between ACEs, PTSD, and overdose needs to be tested with larger samples and more temporal clarity, particularly with greater ability to test differences among Latinx subgroups.

## Data availability statement

The raw data supporting the conclusions of this article will be made available by the authors, without undue reservation.

## Author contributions

CT and LL conceptualized the research question, study design, reviewed the literature, and wrote and edited the initial draft of the manuscript. LL supervised the analytic plan. CT analyzed the data with assistance from LL, MD, and MJL. LL and MJL reviewed various versions of the manuscript. MG, DDJ, and ES oversaw data collection activities. All authors reviewed and approved the final version of the manuscript.
