# Supplementary material for: Adverse childhood experiences are associated with increased overdose risk in predominately Latinx adults seeking treatment for substance use disorders
Source: Front Psychiatry. 2022 Dec 15;13:987085. doi: 10.3389/fpsyt.2022.987085 (PMC9798211; doi:10.3389/fpsyt.2022.987085)
Supplement: Supplementary file 1 [file Data_Sheet_1.pdf]

Supplemental materials

Adverse childhood experiences are associated with increased overdose risk in predominately Latinx adults seeking treatment for substance use disorders

Cynthia A. Tschampl[1], PhD; Melisa Canuto, LICSW[2]; Diliana De Jesús, MA[2]; Melinda D'Ippolito, LICSW, MPH[1]; Micaurys Guzman[2]; Mary Jo Larson, PhD[1]; Emily Stewart, BA[2]; Lena Lundgren, PhD[3]

[1]The Heller School for Social Policy and Management, Brandeis University, Waltham, MA, United States.
[2]Casa Esperanza, Inc., Roxbury, MA, United States.
[3]Graduate School of Social Work, University of Denver, Denver, CO, United States.

Table S1. Bivariate results for correlates of lifetime overdose, reporting PTSD symptoms, and number of ACEs in a sample of treatment seekers in Massachusetts, 2019-2020, n=149

**Independent variable**	**Fisher's exact, 2-sided (P value)**	**Odds Ratio**	**95% CI lower**	**95% CI upper**	**P value**
Bivariate correlates of PTSD positive screen (3-4 symptoms)					
Lifetime use of heroin, years		1.003	0.974	1.032	0.860
Lifetime use of cocaine, years		1.039	1.006	1.074	0.022
Females vs. males	Positive (0.00)				

Bivariate correlates of ACEs scale, 0-10	**Standard-ized Beta coefficient**	**95% CI lower**	**95% CI upper**	**P-value**
Reported 3-4 PTSD symptom categories	1.443	1.246	1.670	0.000
Reported 2-4 PTSD symptom categories	1.586	1.346	1.870	0.000
Lifetime use of heroin, years	0.068	-0.391	0.949	0.412
Lifetime use of cocaine, years	0.060	-0.405	0.868	0.473
Females vs. males	0.237	0.470	2.409	0.004
Latinx ethnicity vs. not	-0.101	-0.033	0.008	0.223
Puerto Rican identity vs. not	0.060	-0.019	0.040	0.474

ACEs, adverse childhood experiences; CI, confidence interval; PTSD, Post Traumatic Stress Disorder

Table S2. Sensitivity analyses using multiple logistic regression models on lifetime overdose, n=147

	**Model 2**[a]			**Model 3**[b]			**Model 4**[c]			**Model 5**[d]		
**Independent variable**	Adjusted odds ratio	95% CI lower	95% CI upper	Adjusted odds ratio	95% CI lower	95% CI upper	Adjusted odds ratio	95% CI lower	95% CI upper	Adjusted odds ratio	95% CI lower	95% CI upper
Total ACE score, 0-10				1.26**	1.06	1.50	1.27**	1.07	1.50	1.30**	1.08	1.56
4 or more ACEs vs. 0-3 ACEs	2.45*	1.00	6.05									
Reported 3-4 PTSD symptom categories	1.78	0.71	4.47				1.48	0.58	3.77	1.5	0.57	3.95
Reported 2-4 PTSD symptom categories				1.66	0.62	4.46						
Reported heroin use vs. not	9.31***	3.11	27.91	9.44***	3.12	28.62	11.11***	3.76	32.83	9.53***	3.00	30.25
Lifetime use of cocaine, years	1.02	0.98	1.06	1.02	0.98	1.06	1.02	0.98	1.06	1.03	0.98	1.07
Puerto Rican identity vs. not	1.70	0.62	4.66	1.74	0.63	4.82				2.07	0.69	6.24
Latinx ethnicity vs. not							0.51	0.14	1.92			
Age, years										0.97	0.92	1.03
Female vs. male	0.99	0.35	2.76	0.86	0.30	2.50	0.56	0.19	1.61	0.65	0.21	2.03
12 or more years of education vs. 0-11 years										2.31	0.94	5.69
Has public assistance income vs. not	1.14	0.49	2.67	1.25	0.54	2.88	1.3	0.55	3.04	1.66	0.64	4.29
Is employed full-time or part-time vs. not										1.68	0.34	8.38
Spent most of the past 30 days on the street/in a shelter, vs. not										1.34	0.57	3.13

ACE, adverse childhood Experiences; CI, confidence interval; PTSD, Post Traumatic Stress Disorder. *p<0.05, **p<0.01, ***p<0.001.

a. Ever overdose = B + Bgender + BPuertoRican + BACEs4 + B3-4PTSDsymp + Bheroin + Bcocaine +Bpublicassist.

b. Ever overdose = B + Bgender + BPuertoRican + BACEscore + B2-4PTSDsymp + Bheroin + Bcocaine +Bpublicassist.

d. Ever overdose = B + Bgender + BHispanicLatino + BACEscore + B3-4PTSDsym + BHeroinYears + Bcocaine +Bpublicassist.

e. Ever overdose = B + Bage + Bgender + BPuertoRican + Bhighschool + Bemployed + Bstreet + BACEscore + B3-4PTSDsym + Bheroin + Bcocaine + Bpublicassist.
